# Serum matrix metalloproteinase 7 (MMP7) is a biomarker of fibrosis in patients with non-alcoholic fatty liver disease

**DOI:** 10.1038/s41598-021-82315-z

**Published:** 2021-02-03

**Authors:** Katharine M. Irvine, Satomi Okano, Preya J. Patel, Leigh U. Horsfall, Suzanne Williams, Anthony Russell, Elizabeth E. Powell

**Affiliations:** 1 Mater Research, The University of Queensland, Brisbane, Australia; 2 Centre for Liver Disease Research, The University of Queensland, Brisbane, Australia; 3 Statistics Unit, QIMR-Berghofer Medical Research Institute, Brisbane, Australia; 4 Institute for Liver and Digestive Health, University College London, London, UK; 5 Department of Gastroenterology and Hepatology, Princess Alexandra Hospital, Brisbane, Australia; 6 Inala Primary Care, Brisbane, Australia; 7 Department of Diabetes and Endocrinology, Princess Alexandra Hospital, Brisbane, Australia; 8 Centre for Health Services Research, The University of Queensland, Brisbane, Australia

**Keywords:** Liver diseases, Biomarkers

## Abstract

Non-alcoholic fatty liver disease (NAFLD) affects 25% of the adult population globally. Since liver fibrosis is the most important predictor of liver-related complications in patients with NAFLD, identification of patients with advanced fibrosis among at-risk individuals is an important issue in clinical practice. Transient elastography is the best evaluated non-invasive method used in referral centres to assess liver fibrosis, however serum-based tests, such as the Enhanced Liver Fibrosis (ELF) score, have a practical advantage as first-line tests due to their wider availability and lower cost. We previously identified matrix metalloproteinase 7 (MMP7) as a serum biomarker of histological advanced fibrosis in a mixed-etiology patient cohort. In this study we aimed to determine the association between MMP7 and fibrosis, assessed by transient elastography, in patients with NAFLD. Serum MMP7 levels were measured in a cohort of 228 patients with NAFLD. Associations between MMP7, liver stiffness measurement (LSM), ELF score and clinical parameters were determined using logistic regression modelling. Serum MMP7 was associated with clinically significant fibrosis (LSM ≥ 8.2), independent of age, gender, BMI and diabetes. The addition of MMP7 significantly improved the diagnostic performance of the ELF test, particularly in patients over the age of 60. Combinations of serum biomarkers have the potential to improve the sensitivity and specificity of detection of advanced fibrosis in at-risk patients with NAFLD. We have demonstrated that serum MMP7 is independently associated with clinically significant fibrosis and improves the diagnostic performance of currently available tests in older patients.

## Introduction

Although non-alcoholic fatty liver disease (NAFLD) affects 25% of the adult population globally^1^, less than 5% of people with NAFLD develop clinically significant liver disease^2^. The most important predictor of liver-related complications and mortality in NAFLD is the presence of advanced fibrosis (Brunt stage 3–4)^3,4^. Therefore, identification of the subset of patients with advanced fibrosis among the immense number of at-risk individuals is an important issue in clinical practice. Transient elastography is the best evaluated non-invasive method used in referral centres to assess the extent of liver fibrosis, with a cut-off < 8.2 kPa providing a high negative predictive value (around 90%) to exclude advanced fibrosis and cirrhosis^5^.

In the primary health care setting, locally available serum biomarkers, such as the Enhanced Liver Fibrosis (ELF) test, have a practical advantage as first-line tests due to their wider availability and lower cost compared with elastography. The ELF test is a commercial panel of markers (tissue inhibitor of matrix metalloproteinase 1 (TIMP1), hyaluronic acid (HA), and the aminoterminal peptide of procollagen III (PIIINP)) focusing on matrix turnover that has good diagnostic accuracy for advanced fibrosis in NAFLD^6^. We have previously shown that in patients with NAFLD (n = 230) there is a moderate positive correlation between ELF test results and LSM, with concordance between the 2 non-invasive biomarkers in 76.5% of cases^7^. Measuring other fibrosis-specific factors along with established serum biomarkers may improve the diagnostic accuracy of these panels. In a discovery study in a mixed-etiology cohort of chronic liver disease patients (n = 432) we identified a novel serum analyte, Matrix Metalloproteinase 7 (MMP7), which, when combined with components of the ELF score, improved the diagnostic accuracy of the ELF model for the identification of advanced fibrosis (Metavir stage 3–4)^8^. In the liver, MMP7 is expressed by hepatocytes, biliary epithelial cells and Kupffer cells^9^, and increased MMP7 expression has been associated with cirrhosis^10^, biliary atresia-associated fibrosis^9^ and HCC migration and invasion^11^. Beyond the liver, MMP7 is also implicated in idiopathic pulmonary fibrosis, where it has been proposed as a biomarker^12^.

Our earlier study of adjunct serum biomarkers of advanced fibrosis was dominated by subjects with chronic hepatitis C (62%), and only 10.6% of the cohort had NAFLD^8^. The aim of this study was to determine whether serum MMP7 identified NAFLD patients with clinically significant fibrosis based on elevated liver stiffness measurements, and to evaluate its diagnostic accuracy in combination with the ELF test.

## Results

### Study population

The demographic and clinical characteristics of the 228 NAFLD patients in this study cohort are presented in Table 1. The mean age of subjects was 56.7 ± 12.3 years, 55.4% were male, with a mean BMI of 34.3 ± 7.8 kg/m^2^, and a high prevalence of type 2 diabetes (82.4%). Median LSM was 6.10 kPa (IQR 4.8–9.2) with a range from 2.5 to 63.9 kPa and required use of the XL probe in 78.4% of subjects. LSM ≥ 8.2 kPa, ≥ 9.5 kPa (consistent with advanced fibrosis) and > 13 kPa (concerning for cirrhosis)^13,14^ were present in 31.1%, 23.7% and 16.2% of patients, respectively. Patients with high LSM had significantly higher markers of liver injury (e.g. liver enzymes), lower albumin and platelets, higher BMI and were more likely to have metabolic syndrome and diabetes (Table 1). Overall, the mean ELF score was 9.30 ± 1.02, with a range from 6.61 to 12.6. An ELF score ≥ 9.8, consistent with severe fibrosis, was present in 25.9% (59/228) of patients (Table 1).

**Table 1:** Study cohort demographics and clinical parameters according to LSM category.

	Total	LSM < 8.2	LSM 8.2–12.9	LSM 13+	p-value
	N = 228	N = 157	N = 34	N = 37	
Male^a^	54.4% (124/228)	53.5% (84/157)	52.9% (18/34)	59.5% (22/37)	0.79
Age (years)^b^	56.7 (12.3)	56.5 (12.4)	54.9 (15.1)	59.2 (8.4)	0.33
BMI, median (IQR)^c^	32.5 (29.1–38.1)	31.2 (28.1–35.8)	36.6 (32.3–40.8)	40.0 (33.5–46.5)	< 0.001
BMI, mean (SD)	34.3 (7.8)	32.2 (6.3)	36.9 (7.0)	40.9 (9.9)	
Girth, mean (SD)^b^	115.4 (18.1)	109.7 (15.3)	124.7 (14.0)	131.0 (19.8)	< 0.001
Metabolic syndrome^a^	83.3% (190/228)	78.3% (123/157)	91.2% (31/34)	97.3% (36/37)	0.009
Diabetes^a^	81.1% (185/228)	75.8% (119/157)	88.2% (30/34)	97.3% (36/37)	0.006
ALT, median (IQR)^c^	33.0 (22.0–54.0)	29.0 (20.0–47.0)	35.0 (27.0–62.0)	50.0 (34.0–65.0)	< 0.001
AST, median (IQR)^c^	24.0 (16.0–36.0)	21.0 (16.0–28.0)	28.0 (17.0–39.0)	38.5 (32.0–66.0)	< 0.001
GGT, median (IQR)^c^	34.0 (21.0–61.0)	27.0 (19.0–43.0)	44.5 (23.0–70.0)	78.0 (51.0–158.0)	< 0.001
Plt, mean (SD)^b^	247.1 (64.1)	253.7 (60.7)	250.3 (55.4)	216.1 (76.9)	0.005
Alb, mean (SD)^b^	41.4 (3.4)	41.8 (3.1)	39.9 (4.0)	40.9 (3.5)	0.007
eGFR, median (IQR)^c^	90.0 (73.0–90.0)	90.0 (73.0–90.0)	89.5 (76.0–90.0)	90.0 (71.0–90.0)	0.82
ELF score, mean (SD)^b^	9.3 (1.0)	9.0 (0.8)	9.4 (0.9)	10.4 (1.0)	< 0.001
ELF ≥ 9.8^a^	25.9% (59/228)	12.7% (20/157)	38.2% (13/34)	70.3% (26/37)	< 0.001
PIIINP (ng/ml), median (IQR)^c^	9.0 (7.0–11.3)	8.1 (6.6–10.2)	9.3 (8.4–12.0)	13.7 (10.7–16.8)	< 0.001
TIMP1 (ng/ml), median (IQR)^c^	237.2 (201.4–276.9)	223.1 (187.9–255.1)	269.0 (225.9–316.5)	306.6 (251.1–358.3)	< 0.001
MMP7 (pg/ml), median (IQR)^c^	923.2 (543.0–1587.9)	764.4 (521.7–1234.2)	1007.9 (810.6–1734.9)	1794.9 (1035.6–2724.3)	< 0.001

Data are shown as mean (SD) or median (IQR) for continuous variables and count (%) for categorical variables.

LSM: liver stiffness measurement, BMI: Body Mass Index, ALT: alanine aminotransferase, AST: aspartate aminotransferase, GGT: gamma-glutamyltransferase , Plt: platelet count (10^9^/L), ELF: enhanced liver fibrosis; HA: hyaluronic acid, TIMP1 tissue inhibitor of metalloprotease 1:, PIIINP: pro-collagen peptide III.

^a^Chi-squared test.

^b^One-way ANOVA.

^c^Kruskal-Wallis test.

### Relationship between MMP7 and fibrosis assessed by LSM

**Figure 1:**
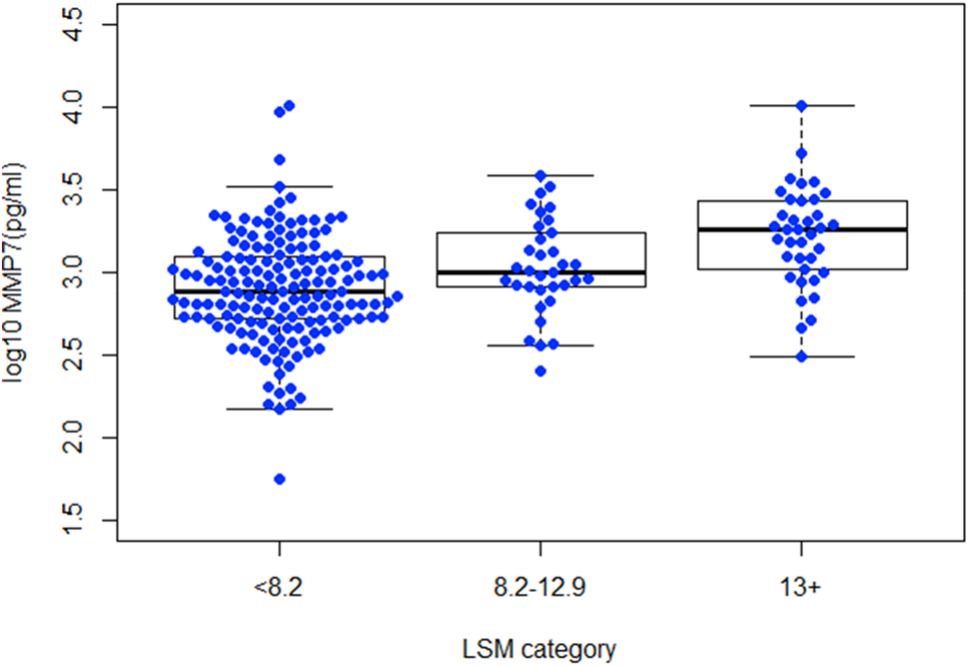
Association between serum MMP7 and LSM. Log serum MMP7 levels in patients with LSM indicative of no significant fibrosis (< 8.2), significant fibrosis (8.2–12.9) and cirrhosis (≥ 13) are shown using box plots with individual points. Upper and lower lines represent 1.5 IQR of the upper and lower quantile, middle line represents median and box represents 25th–75th percentile.

MMP7 was detected in 98.7% of serum samples. Serum MMP7 was positively correlated with LSM (r = 0.35, p < 0.001), with median values of 764.4 (IQR 521.7–1234.2) ng/mL, 1007.9 (IQR 810.6–1734.9) ng/mL and 1794.9 (IQR 1035.6–2724.3) ng/mL, in patients with LSM < 8.2, 8.2–12.9 and > 13 kPa respectively (Fig. 1). Logistic regression modelling revealed that serum MMP7 remained independently associated with clinically significant fibrosis (LSM ≥ 8.2 kPa) after accounting for potential confounding factors (patient age, gender, BMI and diabetes (adjusted Odds Ratio (aOR) 9.65, 95% CI 3.09–30.13) (Table 2).

**Table 2:** Serum MMP7 is independently associated with clinically significant fibrosis (LSM ≥ 8.2 kPa).

	Univariable model	p-value	Multivariable model	p-value
	OR (95% CI)		aOR (95% CI)	
MMP7 (log_10_)	9.83 (3.61, 26.75)***	< 0.001	9.65 (3.09, 30.13)***	< 0.001
Age (per 10 years)	1.04 (0.83, 1.31)	0.72	0.94 (0.69, 1.29)	0.70
**Gender**		0.69		0.95
Female	0.89 (0.51, 1.57)		0.98 (0.50, 1.92)	
BMI	1.13 (1.08, 1.18)***	< 0.001	1.13 (1.08, 1.19)***	< 0.001
Diabetes	4.22 (1.58, 11.23)**	0.004	3.70 (1.14, 11.97)*	0.029

Logistic regression.

*p < 0.05 **p < 0.01 ***p < 0.001.

MMP7 has been reported to be associated with kidney disease^15,16^, and we found a modest negative correlation between MMP7 and estimated glomerular filtration rate (eGFR) (r = − 0.26, p < 0.001). However, the association between MMP7 and clinically significant fibrosis remained strong after further adjustment for eGFR in the model (aOR 9.32, 95% CI 2.99–29.1, p < 0.001), suggesting that the association between MMP7 and fibrosis is independent of eGFR. Further exploratory analysis also revealed a potential interaction between MMP7 and age (p = 0.031 for the interaction model), suggesting the association between MMP7 and LSM may vary with patients’ age. Sub-group analysis by patient age group (< 50, 50–59, 60+) also showed greater aOR with increase in age (aOR (95% CI) 1.91 (0.25, 14.7), 7.36 (0.92, 58.8), 39.4 (5.2, 298.5) respectively. No interactions between MMP7 and other variables were identified.

As a sole biomarker, the diagnostic performance of MMP7 for predicting clinically significant fibrosis (LSM ≥ 8.2 kPa) was moderate (AUROC: 0.70 (95% CI 0.63–0.78), with an optimal cut-off of > 776 ng/mL identified by Youden and Liu methods. By comparison the AUROC of ELF score for the prediction of LSM ≥ 8.2 in this cohort was 0.77 (95% CI 0.70–0.84) (p = 0.1436, De Long’s test). The NAFLD Fibrosis Score (NFS) and the FIB4 score, two so-called ‘simple scores’ calculated from readily-available patient data that may be useful as first-line approaches to stratify fibrosis risk^7^, had AUROCs of 0.71 (95% CI 0.64–0.79) and 0.64 (95% CI 0.56–0.75), respectively. The AUROC of FIB4, but not NFS, was significantly inferior to ELF (p = 0.0024 and p = 0.1656, respectively. De long’s test).

### MMP7 improves the diagnostic performance of the ELF score for predicting clinically significant fibrosis (LSM ≥ 8.2)

ELF score was a significant predictor of clinically significant fibrosis (LSM ≥ 8.2) in this patient cohort (OR 3.15, 95% CI 2.16–4.58, Table 3). We investigated whether the addition of MMP7 improved the diagnostic performance of the ELF score. MMP7 remained significantly associated with clinically significant fibrosis after adjusting for ELF score (p = 0.004), and the reduced Akaike information criterion (AIC) and Bayesian information criterion (BIC) suggested the combined ELF/MMP7 model was a more parsimonious model than ELF alone. The category free Net Reclassification Improvement (NRI) was 0.268 for LSM ≥ 8.2 and 0.261 for LSM < 8.2 (total NRI 0.53), indicating a 26.8% net improvement in the prediction of clinically significant fibrosis and a 26.1% net improvement in the prediction of non-significant fibrosis. When cut-offs were chosen to achieve 90% specificity in these models, sensitivity, PPV and NPV improved from 43.7 to 52.1%, from 67.4 to 71.2%, and from 78.0 to 80.7% respectively (Table 3). In real terms, of 71 patients with LSM ≥ 8.2 the number of patients correctly predicted increased from 31 to 37 (Supplementary  Table 1).

**Table 3:** Performance of models with ELF and ELF + MMP7 for the prediction of LSM > 8.2.

	ELF	ELF + MMP7
	OR (95% CI)	aOR (95% CI)
ELF score	3.15 (2.16,4.58)***	2.81 (1.91,4.14)***
MMP7 (log_10_)		5.15 (1.75,15.2)**
**Model performance**		
AUROC (95% CI)	0.77 (0.70–0.84)	0.79 (0.72–0.86)
McFadden (adjusted)	0.157	0.183
AIC	238.5	231.0
BIC	245.4	241.3
**Category-free NRI**		
NRI for LSM 8.2+ (SE)	0.268 (0. 114)	
NRI for LSM < 8.2 (SE)	0.261 (0.077)	
Total NRI (SE)	0.529 (0.138)	
IDI (SE)	0.0405 (0.012)**	
**At 90% specificity**		
Sensitivity (%)	43.7	52.1
PPV (%)	67.4	71.2
NPV (%)	78.0	80.7
LR+	4.57	5.45
LR−	0.62	0.53

Logistic regression.

AUROC: area under the receiver operating curve, AIC: Akaike information criterion, BIC: Bayesian information criterion, NRI: net reclassification improvement, IDI: integrated discrimination improvement, PPV: positive predictive value, NPV: negative predictive value, LR+: positive likelihood ratio, LR−: negative likelihood ratio.

**p < 0.01, ***p < 0.001.

### MMP7 may be a valuable biomarker for the prediction of clinically significant fibrosis in older people

Since we identified a potential interaction between age and MMP7 in the multivariable logistic regression model (age/MMP7 interaction p = 0.031), we performed a sub-group analysis to further compare between MMP7 and ELF in predicting clinically significant fibrosis in patients older (or younger) than 60. Age 60 was chosen because earlier subgroup analysis indicated greater association between MMP7 and LSM ≥ 8.2 in patients above 60. The data suggest the ELF model does not perform as well in patients over 60 compared to the younger age group. Comparing the performance of the ELF and combined ELF + MMP7 models in each age group (< 60, 60+) indicated that MMP7 had little improvement on the ELF model in the younger age group but significantly improved upon the ELF model in the older age group (Table 4). The increased standardised coefficient of MMP7 in the older age group indicates that MMP7 is a stronger predictor of LSM ≥ 8.2 kPa than the ELF score in the older age group.

**Table 4:** Performance of ELF and ELF + MMP7 models for predicting LSM ≥ 8.2 by age group.

	Age < 60 (n = 124)			Age 60+ (n = 104)		
	ELF	ELF + MMP7		ELF	ELF + MMP7	
	OR (95% CI)	aOR (95% CI)	${\beta }_{st}$	OR (95% CI)	aOR (95% CI)	${\beta }_{st}$
ELF score	4.45 (2.37, 8.33)***	4.33 (2.26, 8.30)***	0.62	2.95 (1.64, 5.32)***	3.15 (1.63, 6.10)**	0.40
MMP7 (log_10_)		1.27 (0.28, 5.77)	0.035		42.1 (5.73, 309.5)***	0.48
**Model performance**						
AUROC (95% CI)	0.79 (0.69–0.89)	0.79 (0.69–0.89)		0.74 (0.63–0.84)	0.83 (0.75–0.91)	
McFadden (adjusted)	0.215	0.202		0.090	0.216	
AIC	117.3	119.3		121.0	104.1	
BIC	123.0	127.7		126.2	112.1	
**At 90% specificity**						
Sensitivity (%)	47.2	50.0		28.6	48.6	
PPV (%)	68.0	69.2		58.8	70.8	
NPV (%)	80.8	81.6		71.2	77.5	

Logistic regression.

βst: standardised coefficient, AUROC: area under the receiver operating curve, AIC: Akaike information criterion, BIC: Bayesian information criterion, PPV: positive predictive value, NPV: negative predictive value, LR+ : positive likelihood ratio, LR−: negative likelihood ratio.

**p < 0.01, ***p < 0.001.

### Contribution of MMP7 and the ELF test analytes to predicting clinically significant fibrosis (LSM ≥ 8.2)

Since ELF is a composite score calculated from serum levels of 3 biomarkers (HA, PIIINP and TIMP1), we analysed the association of individual serum analytes, including MMP7, with LSM-assessed fibrosis. There were significant positive correlations between MMP7 and ELF score (r = 0.30) and all individual biomarkers (HA r = 0.26, PIIINP r = 0.27, TIMP1 r = 0.34). The ELF score components were highly correlated among themselves to a greater degree than between MMP7 and any component. The ELF score was highly correlated with patient age (r = 0.48), with a particularly high correlation between HA and age (r = 0.53), whereas there was only a modest correlation (r = 0.25) between MMP7 and age. A multivariable model with individual biomarkers was used to investigate relative strength of association between LSM ≥ 8.2 and these biomarkers. The standardised coefficients of MMP7, HA, PIIINP and TIMP1 in the model predicting LSM ≥ 8.2 were 0.16, 0.20, 0.10 and 0.44 (0.19, 0.36, 0.028 and 0.36 when adjusted for gender, age, BMI and Diabetes), indicating that TIMP1 is the strongest predictor of LSM ≥ 8.2 and PIIINP is the weakest predictor in a multivariable model in this cohort (Table 5). Consistent with the results in Table 4, in patients younger than 60, MMP7 did not improve upon the combination of HA, PIIINP and TIMP1 for predicting LSM ≥ 8.2. However, MMP7 had the highest standardised co-efficient of all serum analytes in the older age group, suggesting MMP7 is the strongest predictor of LSM ≥ 8.2 in patients older than 60 (Table 5). TIMP1 and HA were the strongest predictors of LSM ≥ 8.2 in patients < 60. MMP7 was also a strong predictor of LSM ≥ 9.5 (advanced fibrosis) and LSM ≥ 13.0 (concerning for cirrhosis) in older patients (Supplementary Table 2). PIIINP also tended to be a stronger predictor of fibrosis in older patients, whereas HA was more useful in patients younger than 60 (Table 5 and Supplementary Table 2).

**Table 5:** Standardised coefficients of serum biomarkers for the prediction of clinically significant fibrosis (LSM ≥ 8.2) using multivariable logistic regression models by age above or below 60.

	Whole cohort		Age < 60		Age 60+	
	aOR (95% CI)	${\beta }_{st}$	aOR (95% CI)	${\beta }_{st}$	aOR (95% CI)	${\beta }_{st}$
MMP7	1.65 (1.00, 2.71)*	0.16	1.00 (0.48, 2.09)	0.001	4.21 (1.69, 10.5)**	0.38
HA	1.74 (1.12, 2.69)*	0.20	3.73 (1.65, 8.45)**	0.41	1.72 (0.84, 3.52)	0.16
PIIINP	1.94 (0.61, 6.14)	0.10	0.81 (0.17, 3.95)	− 0.030	3.84 (0.55, 26.8)	0.18
TIMP	73.2 (10.8, 497.7)***	0.44	183.9 (10.3, 3270.6)***	0.50	57.2 (2.67, 1228.6)**	0.35

All biomarker concentrations in natural log.

*p < 0.05, **p < 0.01, ***p < 0.001.

## Discussion

Non-invasive serum biomarkers to discriminate advanced from nonadvanced fibrosis in patients with NAFLD are urgently required. Although currently available non-invasive tests have acceptable diagnostic performance, the misclassification rate is at least 20% and occurs primarily in patients with histological advanced fibrosis and “false-negative” biomarkers^17^. In our cohort of patients with NAFLD, we found that the addition of serum MMP7 improved the diagnostic performance of the ELF score, increasing the sensitivity by 8.5% in the total cohort and by 20% in patients over 60, at a 10% false positive rate (90% specificity). Given the large proportion of the population with, or at risk of, NAFLD, an improvement of this magnitude in diagnostic sensitivity would clearly be clinically significant.

Few studies have examined the performance characteristics of the ELF test with elastography in patients with chronic liver disease, and the different thresholds chosen for each modality make comparison challenging. In a study of 283 subjects (74.9% HCV and/or HIV-infected), an ELF test cut-off of 10 had high specificity (98.9%, 95% CI 96.7–99.8%) with an AUROC of 0.80 (95% CI 0.749–0.845) for advanced fibrosis using magnetic resonance elastography (MRE) as the reference methodology, although sensitivity was poor^18^. In another study of 181 treatment-naïve patients with chronic HCV, an ELF test cut-off of 9.8 had 77.8% sensitivity and 67.1% specificity for predicting advanced fibrosis determined by LSM ≥ 9.6 kPa, although the ELF test components were measured using non-proprietary assays on an alternative platform^19^. A direct comparison of non-invasive tests in alcohol-related liver disease (n = 289), identified 26 (9%) patients with discordance between the ELF test and LSM^20^. In patients with ELF < 10.5 but LSM ≥ 15 kPa, 9 of 12 patients had advanced fibrosis; whereas in patients with ELF ≥ 10.5 but LSM < 15 kPa, 14 of 14 patients did not have advanced fibrosis^20^. We have previously shown concordance between ELF test ≥ 9.8 and LSM ≥ 8.2 kPa in 76.5% of a high-risk cohort of patients with NAFLD^7^. In the latter study, patients with discordant elevated ELF tests were significantly older^7^, suggesting that age may impact on ELF score discrimination. Importantly, in a more recent study, ELF test specificity for advanced fibrosis decreased from 73% (overall) to less than 60% among NAFLD patients ≥ 65 years^17^, highlighting the fact that age is potentially a significant confounder for the ELF test.

Our data support the potential for improvement in detection of advanced fibrosis using further combinations of biomarkers, particularly in patients older than 60. Although the diagnostic performance of MMP7 alone is moderate, the need for combinations of biomarkers has long been recognized. The independent association with advanced fibrosis and model improvement suggest that MMP7 may be detecting a distinct pathological process. In infants with biliary atresia (a progressive fibroinflammatory cholangiopathy), serum MMP7 is emerging as a diagnostic and prognostic biomarker^21^ and has been proposed as a potential therapeutic target^22,23^. In liver sections from children with biliary atresia, MMP7 expression was localized in the bile duct epithelium and proliferating ductules^24^, and in an experimental model of biliary injury, MMP7 was shown to modulate tissue injury and inflammation^22^. However, studies examining the role of MMP7 in other chronic liver diseases remain limited. A study utilising laser capture microdissection and RNA Sequencing to analyse areas of ductular reaction within fibrotic scars from patients with biliary (primary sclerosing cholangitis) compared to hepatocellular (HCV) fibrosis demonstrated enriched *MMP7* expression in the biliary cases, further suggesting an association with biliary activation^25^. In a study of 320 patients with hepatocellular carcinoma with or without cirrhosis, a nonsynonymous single‐nucleotide polymorphism in the MMP‐7 gene was associated with the presence of cirrhosis^26^ although this observation does not appear to have been replicated. In a small study (n = 80 patients with NAFLD and 59 “healthy controls”), serum MMP7 levels were significantly different between patients with NAFLD and controls but were not correlated with histological fibrosis^27^. Interestingly the farnesoid X receptor (FXR) has been shown to act as a transcriptional repressor of MMP7 in colon cancer cells, and an inverse relationship has been observed between FXR and MMP7 expression^28^. It will be important to determine whether this regulatory pathway also occurs in human liver cells, as the FXR agonist obetacholic acid is being evaluated for the treatment of NAFLD^29^.

Measuring serum biomarkers may be confounded by the presence of systemic disease processes and may not be specific for liver injury. Previous studies have shown elevated serum and/or urine MMP7 levels in patients with kidney disease^30^, particularly in people with type 2 diabetes^15,16^. In addition, a decrease in serum MMP7 levels has also been shown following treatment of diabetic kidney disease with a sodium–glucose cotransporter 2 inhibitor, likely reflecting an improvement in extracellular matrix turnover and fibrosis^31^. In our patient cohort, the association between MMP7 and liver fibrosis was independent of T2D and impaired renal function (assessed by eGFR), common comorbidities in patients with NAFLD. However future studies will also need to address the impact of specific pharmacotherapy, as this was outside the scope of the current study.

Strengths of our study include the independent validation of our previous finding of an association between MMP7 and fibrosis^8^, in a separate, well-characterised cohort of patients with NAFLD recruited from primary care and diabetes clinics. Our data add to the current literature supporting a role for MMP7 as a biomarker of advanced fibrosis, particularly in older patients. Key limitations of the study are the lack of histological assessment of fibrosis and the relatively small size of the cohort. Nonetheless, elastography is well established in hepatology centres for determining advanced fibrosis or cirrhosis. The use of the non-invasive liver stiffness measurement as the determinant of fibrosis in this study necessitated selection of a threshold for significant fibrosis. Whilst appropriate thresholds for LSM are still under discussion in the literature, thresholds of 6.5–8.2 kPa have been reported to have approximately 90% sensitivity in excluding stage 3–4 fibrosis^14,32^. We conservatively selected a threshold at the upper end of reported values to positively identify fibrosis. Moreover, LSM’s positive predictive value is relatively modest; which suggests the strength of the association between MMP7 and liver fibrosis may be conservative. As non-invasive serum tests permeate in the community, knowledge of the performance characteristics of these liver tests in comparison with elastography will be increasingly important.

In conclusion, we have demonstrated that serum MMP7 levels are independently associated with clinically significant fibrosis in patients with NAFLD; and that MMP7 is a particularly strong predictor of fibrosis in patients older than 60. Refining the sensitivity and specificity of non-invasive biomarkers of fibrosis in this patient cohort could lead to significant clinical and economic gains, given the size of the population at risk.

## Methods

### Subjects

This is a retrospective study of patients identified with NAFLD between October 2015 and August 2017 who attended the liver clinic at the Princess Alexandra Hospital, Brisbane for clinical assessment (n = 252). The source population has previously been described^7^. Informed written consent was obtained from each eligible patient and the protocol was approved by the Metro South Health and The University of Queensland Human Research Ethics Committees (HREC/15/QPAH/301; UQ2015001047). All methods were performed in accordance with relevant guidelines and regulations.

### Clinical assessment

Clinical assessment included anthropometric measurements, routine haematological, biochemical and serological tests, ELF test, LSM and liver ultrasound. Transient elastography was performed using FibroScan technology (Echosens, Paris, France) as previously described^7^. For the purposes of this study we used a cut-off value of ≥ 8.2 kPa for clinically significant fibrosis, for both standard M and XL probes^7^.

### Serum MMP7 measurement

An aliquot of fasted serum collected at clinical assessment was stored at − 80 °C prior to analysis. Serum MMP7 level was determined in 228 of the 230 patients from the original study who had LSM meeting quality criteria, using a sensitive sandwich enzyme-linked immunosorbent assay (RnD Systems, Australia). Two patients were not included in this study as insufficient serum was available. Sera were diluted 1:5 in the manufacturer’s recommended diluent for analysis. All samples were assayed in duplicate and the mean serum MMP7 level was analysed. MMP7 was below or above the limit of detection in 2 and 1 samples, respectively. MMP7 values in these samples were imputed by minimum and maximum detectable values.

### Statistical analyses

Patient characteristics were summarised with mean (standard deviation, SD) or median (interquartile range, IQR) for normally distributed or skewed continuous variables, respectively, and frequency (%) for categorical variables. Characteristics of patients in different LSM categories (< 8.2, 8.2–12.9, > 13) were compared using one-way ANOVA or Kruskal–Wallis test for normally distributed or skewed continuous variables, respectively, and Chi-squared test for categorical variables. Association between MMP7 and clinically significant fibrosis, defined as LSM ≥ 8.2 kPa, was examined using a binary logistic regression model. Multivariable modelling was used to account for potential confounding effects of pre-selected variables including gender, age, BMI and diabetes. Interactions between MMP7 and each of these factors were also examined. The diagnostic performance of ELF alone and ELF + MMP7 models to predict LSM ≥ 8.2 kPa were described using AUROC, Akaike information criterion (AIC), Bayesian Information Criterion (BIC), category free Net Reclassification Index (NRI)^33^ and Integrated Discrimination Improvement (IDI)^34^ based on a binary logistic regression model. Sensitivity, PPV and NPV were calculated using cut-offs which achieved 90% specificity (10% false positive rate). To examine the relative importance of individual serum biomarkers or ELF score in a multivariable model, estimated standardised coefficients were also presented. All analyses were performed using Stata 15.0 (StataCorp. 2017. Stata Statistical Software: Release 15. College Station, TX: StataCorp LLC) and R statistical package (v3.6.1; R Core Team, 2019). The level of statistical significance was set at 0.05.

## Supplementary Information

Supplementary Information.
