# Supplementary material for: Serum matrix metalloproteinase 7 (MMP7) is a biomarker of fibrosis in patients with non-alcoholic fatty liver disease
Source: Sci Rep. 2021 Feb 3;11:2858. doi: 10.1038/s41598-021-82315-z (PMC7858627; doi:10.1038/s41598-021-82315-z)
Supplement: Supplementary file 1 — Supplementary Information. [file 41598_2021_82315_MOESM1_ESM.docx]

**Supplementary Information**

**Serum Matrix Metalloproteinase 7 (MMP7) is a biomarker of fibrosis in patients with non-alcoholic fatty liver disease.**

Katharine M Irvine^1,2#*^, Satomi Okano^3#^, Preya J Patel^2,4^, Leigh U Horsfall^2,5^, Suzanne Williams^6^, Anthony Russell^7,8^, Elizabeth E Powell^2,5*^

^1^ Mater Research, The University of Queensland, Brisbane, Australia

^2^ Centre for Liver Disease Research, The University of Queensland, Brisbane, Australia

^3^ Statistics Unit, QIMR-Berghofer Medical Research Institute, Brisbane Australia

^4^ Institute for Liver and Digestive Health, University College London, London

^5^ Department of Gastroenterology and Hepatology, Princess Alexandra Hospital, Brisbane, Australia

^6^ Inala Primary Care, Brisbane, Australia

^7^ Department of Diabetes and Endocrinology, Princess Alexandra Hospital, Brisbane, Australia

^8^ Centre for Health Services Research, The University of Queensland, Brisbane, Australia

# Contributed equally

* Corresponding authors: Dr Katharine Irvine [Katharine.irvine@uq.edu.au](mailto:Katharine.irvine@uq.edu.au) (ORCID: 0000-0002-6716-1605), Professor Elizabeth Powell [e.powell@uq.edu.au](mailto:e.powell@uq.edu.au)

**Supplementary table 1**. Contingency table illustrating the diagnostic accuracy of the ELF and ELF+MMP7 models compared to LSM ≥8.2 (gold standard) at 90% specificity.

|  | **LSM** |  |  |
| --- | --- | --- | --- |
| **ELF** | **+ (≥8.2)** | **- (<8.2)** | Total |
| + | **31** | 15 | **46** |
| - | 40 | 142 | 182 |
| Total | 71 | 157 | 228 |
| **ELF + MMP7** | **+ (≥8.2)** | **- (<8.2)** | Total |
| + | **37** | 15 | **52** |
| - | 34 | 142 | 176 |
| Total | 71 | 157 | 228 |

**Supplementary Table 2**. Standardised coefficients of biomarkers for the prediction of LSM ≥9.5 and LSM ≥13 by age group (<60, 60+) using multivariable logistic regression models.

| **LSM** ≥ **9.5** |  | | | |
| --- | --- | --- | --- | --- |
|  | **Age < 60** | | **Age 60 +** | |
|  | aOR (95% CI) | $\beta_{st}$ | aOR (95% CI) | $\beta_{st}$ |
| MMP7 | 1.13 (0.48,2.69) | 0.034 | 2.75 (0.99,7.65) | 0.24 |
| HA | 4.54 (1.79,11.55)** | 0.45 | 1.91 (0.85,4.29) | 0.17 |
| PIIINP | 0.87 (0.15,5.16) | -0.019 | 14.27 (1.51,134.68)* | 0.31 |
| TIMP | 173.67 (6.90,4372.97)** | 0.47 | 167.94 (5.28,5342.68)** | 0.39 |
| **LSM** ≥ **13.0** |  | | | |
|  | **Age < 60** | | **Age 60 +** | |
|  | aOR (95% CI) | $\beta_{st}$ | aOR (95% CI) | $\beta_{st}$ |
| MMP7 | 1.40 (0.49,4.06) | 0.091 | 5.15 (1.55,17.1)** | 0.39 |
| HA | 6.95 (2.17,22.2)** | 0.56 | 2.32 (0.93,5.80) | 0.22 |
| PIIINP | 1.41 (0.17,11.4) | 0.046 | 17.22 (1.47,202.17)* | 0.33 |
| TIMP | 21.4 (0.71,647.6) | 0.28 | 19.22 (0.61,606.61) | 0.23 |

* p <0.05 ** p <0.01 *** p < 0.001
